# Prognostic value of angiopoietin-2 for patients with coronary heart disease after elective PCI

**DOI:** 10.1097/MD.0000000000014216

**Published:** 2019-02-01

**Authors:** Wen Jian, Lang Li, Xiao-Min Wei, Cheng-Qiang Wu, Chun Gui

**Affiliations:** a Department of Cardiology, The First Affiliated Hospital of Guangxi Medical University; b Guangxi Key Laboratory Base of Precision Medicine in Cardio-Cerebrovascular Diseases Control and Prevention; c Guangxi Clinical Research Center for Cardio-Cerebrovascular Diseases, Nanning; d Department of Cardiology, Gongren Hospital of Wuzhou, Wuzhou; e Department of Cardiology, The First Affiliated Hospital, Guangxi University of Chinese Medicine, Nanning, Guangxi, People's Republic of China.

**Keywords:** angiopoietin-2, biomarker, cardiovascular events, coronary heart disease, percutaneous coronary intervention

## Abstract

Patients with coronary heart disease (CHD) frequently have cardiovascular complications after undergoing PCI. Angiopoietin-2 (Ang-2) is an important proangiogenic factor that also plays an important role in atherosclerosis. This study aimed to evaluate the value of Ang-2 in predicting cardiovascular events after elective PCI.

This prospective study enrolled 97 patients with CHD who underwent elective PCI from 2013 to 2014. Blood samples were collected in the first morning after admission and within 24 to 48 h after PCI. The primary endpoint was cardiovascular events, defined as a composite of cardiac death, nonfatal myocardial infarction/repeat revascularization, readmission for severe deterioration of angina and readmission for new onset heart failure. Based on the median level of pre-PCI or post-PCI Ang-2, the patients were divided into a low level group and a high level group.

During the whole follow-up period (mean, 53 ± 13 months), Kaplan–Meier curves of cardiovascular events showed that there was no significant difference between the two pre-PCI groups (*χ*^2^ = 2.22, *P* = .137, and log-rank test) or the two post-PCI groups (*χ*^2^ = 2.83, *P* = .093, and log-rank test). However, in a multivariable Cox regression model, landmark analysis showed that the patients in high level group of post-PCI, not pre-PCI, were associated with remarkable higher risks of cardiovascular events compared to the low level group during the first 1.5 years of follow-up (adjusted HR = 9.99, 95%CI = 1.99–50.13, *P* = .005). However, that was of no significance from 1.5 years to maximum follow-up years (adjusted HR = 0.82, 95%CI = 0.26–2.59, *P* = .733).

High Ang-2 levels of post-PCI can predict the occurrence of cardiovascular events in the short to medium term.

## 1 Introduction

Coronary heart disease (CHD) is the leading cause of death worldwide and affects millions of people. Percutaneous coronary intervention (PCI) is widely accepted as an effective and safe treatment for CHD. However, subsequent occurrences of cardiovascular events after PCI are of great concern. There are many factors that can lead to the occurrence of cardiovascular events, such as in-stent restenosis, new-onset atherosclerosis, and deterioration of cardiac function. Previous studies have shown that the inflammatory response plays an important role in the progression of atherosclerosis and plaque destabilization.^[1]^ Angiopoietin-2 (Ang-2), apart from its role in angiogenesis, is also an important regulator of inflammation.^[2]^ Its negative prognostic role in many cardiovascular diseases, such as cardiac shock, chronic heart failure, and aortic aneurysm has been recently reported.^[3–5]^ In addition, even in the general population or among those without cardiovascular disease, high levels of circulating Ang-2 can predict the occurrence of major adverse cardiovascular events (MACE).^[6,7]^ However, the prognostic role of Ang-2 after elective PCI has not been reported until now.

The endothelium functions as a barrier that can regulate the permeability of inflammatory cells, and is controlled by the angiopoietin (Ang)-Tie ligand–receptor system.^[8]^ Angiopoietin-1 (Ang-1), which binds to the Tie-2 receptor, is able to stabilize endothelial and vascular structures.^[9]^ Conversely, Ang-2 is released from Weibel–Palade bodies by several stimuli and acts as a natural antagonist of Ang-1 through interfering with Ang-1-Tie-2 signaling.^[10]^ High levels of Ang-2 can lead to impaired integrity of the endothelium and promote monocyte adhesion and migration by sensitizing endothelial cells towards tumor necrosis factor-α (TNF-α).^[11]^ In addition, Ang-2 is closely associated with the progression of atherosclerosis.^[12]^ In a large population-based sample, circulating Ang-2 levels are found to be associated with the number of carotid plaques.^[13]^ Biopsies taken from human atherosclerotic arteries show that Ang-2 is abundantly expressed in advanced lesions.^[14]^ A high microvessel density, which can lead to hemorrhage and plaque rupture, is associated with plaque Ang-2 levels.^[15]^ In vivo, angiopoietin-2 blocking antibodies are able to reduce early atherosclerotic plaque development in mice.^[16]^

Given the above information, high levels of Ang-2 may predispose patients to cardiovascular events after PCI. Based on our recent research, which showed that serum concentrations of Ang-2 are elevated in CHD patients and decrease significantly after PCI,^[17]^ this study aimed to determine whether serum Ang-2 levels of pre- or post-PCI could serve as independent predictors of cardiovascular events in patients with CHD after elective PCI.

## 2 Methods

### 2.1 Patients and study design

This prospective study enrolled 97 patients with CHD who underwent elective PCI from 2013 to 2014. The following exclusion criteria were applied: acute phase of myocardial infarction, valvular heart disease, systemic infectious diseases, severely impaired renal function, tumor, and autoimmune diseases. The study protocol was reviewed and approved by the Human Research Ethics Committee of the First Affiliated Hospital of Guangxi Medical University, China. The baseline data of patients were collected on the first day after admission. All patients received standard medical treatment for their clinical conditions and used drug-eluting stents. Written informed consents were obtained from all patients. All participants were contacted by telephone periodically and their medical records were followed up regularly until May 2018. The primary endpoint was cardiovascular events, which were defined as a composite of cardiac death, nonfatal myocardial infarction/repeat revascularization, readmission for severe deterioration of angina, and readmission for heart failure. Secondary endpoints included individual components of the primary endpoint. Severe deterioration of angina was defined as recurrent severe angina at rest or at minimum exertion compared to the discharged condition after PCI. All patients with recurrent severe angina were subsequently admitted to hospital and underwent repeat coronary angiography to determine if revascularization was necessary.

### 2.2 Laboratory measurements

Venous blood samples for Ang-2 level assessment were collected in the first morning after admission and within 24 to 48 h after PCI. All blood samples were collected into test tubes and were kept without an anticoagulant for 0.5 h at room temperature, and were then centrifuged at 5000 rev/min for 5 min. The serum supernatant was removed and stored at −80°C until it was used for analysis. Ang-2 concentrations in the blood samples were measured using enzyme-linked immunosorbent assay kits according to the manufacturer's instructions (RayBiotech, Inc, Norcross, GA). In order to minimize measurement errors, samples were assayed in duplicate. Data were extracted using SigmaPlot (Systat Software Inc, San Jose, CA).

### 2.3 Statistical analysis

Continuous variables are presented as mean ± SD and were compared using the Student *t* test or the Mann–Whitney test, as appropriate. Categorical variables were compared using the chi-square or the Fisher exact test. Kaplan–Meier curves were constructed using log-rank tests for the primary endpoint. Multivariate analysis was performed using Cox regression analysis. Landmark analyses were performed according to a landmark point of 1.5 years, with the hazard ratio calculated separately for events that occurred up to 1.5 years after PCI and for events that occurred between 1.5 years after PCI and the end of the follow-up period. The results are presented as the hazard ratio (HR) and relative risk with 95% confidence. Two-sided *P* values of <.05 are considered statistically significant. All analyses were performed by an independent statistician from Guangxi Medical University with the use of Stata software, version 14.0.

## 3 Results

**Figure 1:**
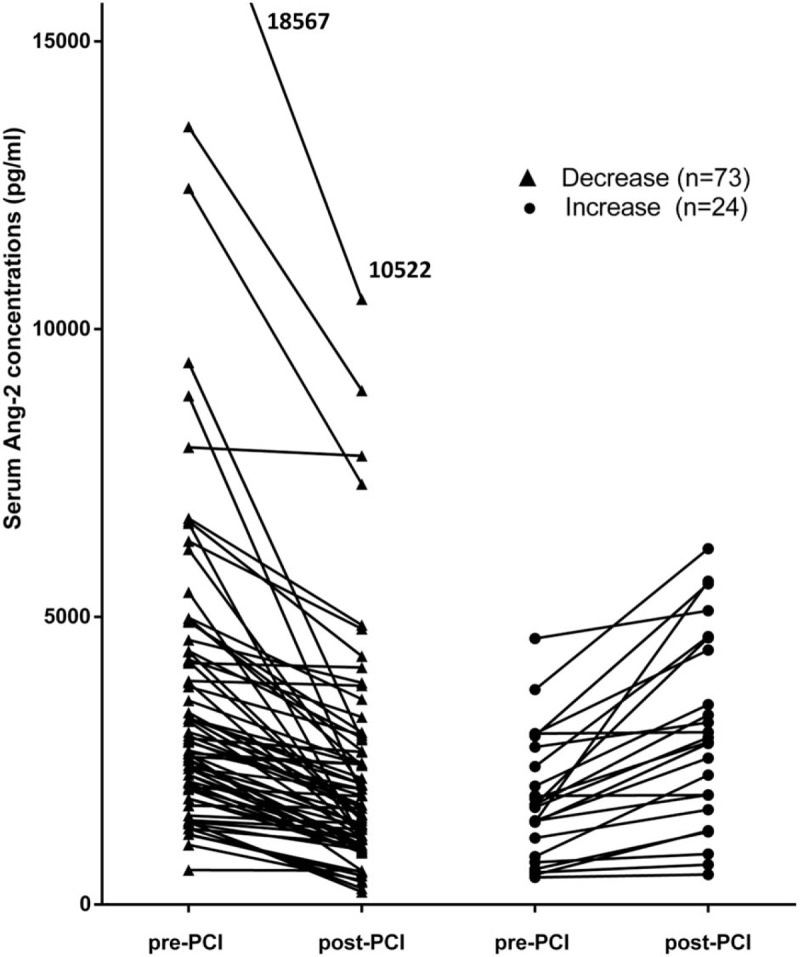
Effect of elective PCI on serum angiopoietin-2 levels (pre-PCI vs. post-PCI – paired samples). PCI = percutaneous coronary intervention.

Post-PCI serum Ang-2 concentrations were significantly lower than pre-PCI concentrations (post-PCI serum Ang-2 concentration: 2385.42 ± 1880.95 pg/ml; pre-PCI serum Ang-2 concentration: 3255.78 ± 2787.51 pg/ml; *P* < .001). As shown in Figure 1, PCI resulted in a decrease in serum Ang-2 levels in most patients (73 of 97, average reduction 40%). In 24 patients there was an increase in serum Ang-2 levels after PCI (average rise 75%). Based on the median level of pre-PCI Ang-2 concentration (2523.86 pg/ml) or post-PCI Ang-2 concentration (1888.43 pg/ml), patients were divided into two subgroups (a low level group and a high level group). There was no clinical evidence of interventional complications after PCI. All patients took medicine on time after discharge.

### 3.1 Characteristics of the two subgroups based on the median level of post-PCI Ang-2

The baseline data are summarized in Table 1. Patients were classified according to the median level of post-PCI Ang-2. No significant difference was found between the groups in terms of age, gender, BMI, smoking, comorbidities of hypertension, diabetes, hyperlipidemia, classification of CHD, systolic and diastolic blood pressure, LVDD, LVSD, LVEF, troponin I, serum creatinine, lesion vessel numbers, treated vessel numbers, and medication at admission. Thirty patients underwent the complete revascularization at index procedure (high level group: *n* = 13; low level group: *n* = 17; *P* = .42). Of note, there were more patients with hyperlipidemia in the low level group than in the high level group, although this difference was not significant.

**Table 1:**
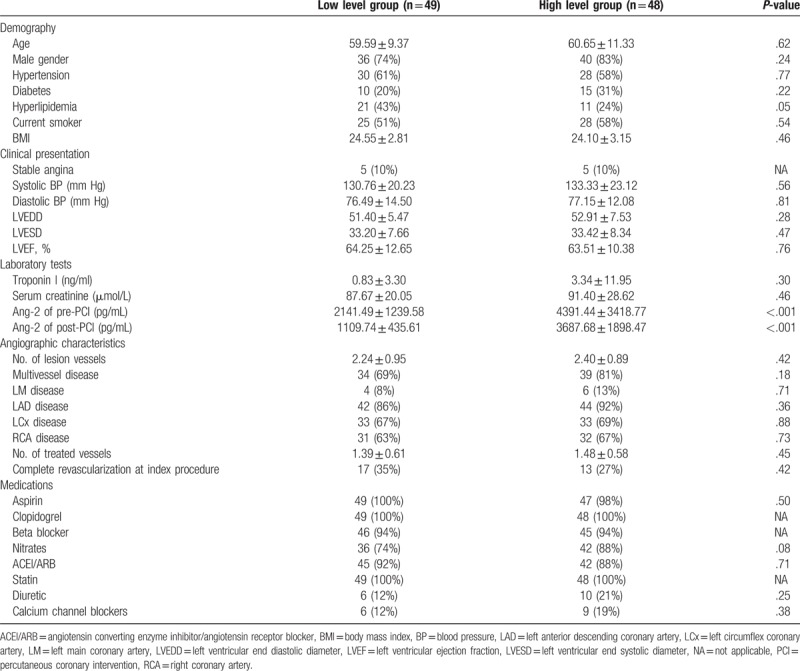
Baseline characteristics of two study subgroups based on the Ang-2 median level of post-PCI (1888.43 pg/ml).

### 3.2 Clinical outcome of the entire follow-up period

**Figure 2:**
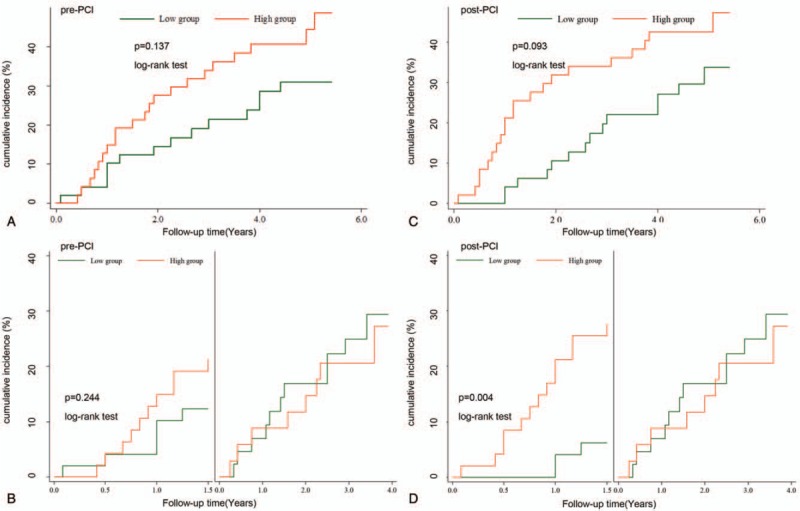
Kaplan–Meier curves for the cumulative incidence of cardiovascular events during the whole follow-up period (A) and its Landmark analyses according to a landmark point at 1.5 years (B) between the low level group and the high level group based on the Ang-2 median level of the pre-PCI (2523.86 pg/ml). Kaplan–Meier curves for the cumulative incidence of cardiovascular events during the whole follow-up period (C) and its Landmark analyses according to a landmark point at 1.5 years (D) between the low level group and the high level group based on the Ang-2 median level of the post-PCI (1888.43 pg/ml). PCI = percutaneous coronary intervention.

During the follow-up period (mean: 53 ± 13 months), 35 adverse cardiovascular events occurred (cardiac death: *n* = 0; nonfatal myocardial infarction/repeat revascularization: *n* = 17; readmission for severe angina: *n* = 25; readmission for heart failure: *n* = 8). In patients who had the first readmission for severe angina after elective PCI, 40% of them (10/25) underwent the repeat revascularization. Although the frequency of cardiovascular events of the high level pre-PCI group (21/48, 43.8%) were higher than that of the low level pre-PCI group (14/49, 28.6%), the Kaplan–Meier curves showed that there was no significant difference between the two pre-PCI groups (*χ*^2^ = 2.22, *P* = .137, Fig. 2A). The results of the Kaplan–Meier curves for the frequency of cardiovascular events of the two post-PCI groups were similar (*χ*^2^ = 2.83, *P* = .093, Fig. 2C). However, as shown in Table 2, after adjusting for the baseline covariates of age, gender, BMI, hyperlipidemia, LVEF, serum creatinine, classification of CHD, and lesion vessel numbers, multivariate Cox regression analysis revealed that the high Ang-2 level of post-PCI was an independent predictor of cardiovascular events (adjusted HR = 2.33, 95%CI = 1.04–5.18, *P* = .039), while the high Ang-2 level of pre-PCI was not found to be an independent predictor (adjusted HR = 2.02, 95%CI = 0.92–4.46, *P* = .078). Additionally, the results of secondary endpoints are also shown in Table 2.

**Table 2:**
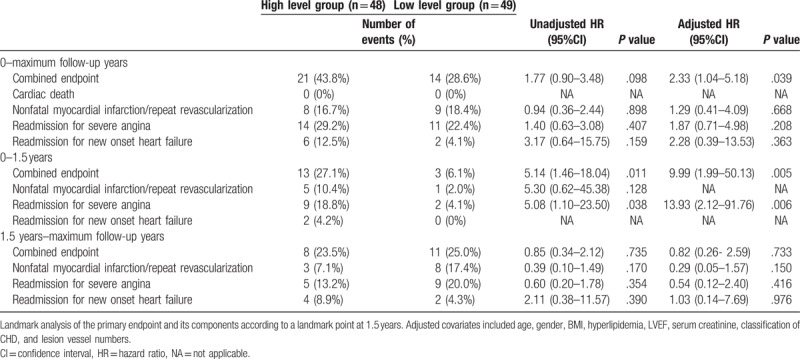
Results of the univariate and multivariate cox regression models investigating the prognostic value of increased Ang-2 concentrations of post-PCI (above 1888.43 pg/ml).

### 3.3 Landmark analyses according to a landmark point of 1.5 years

Landmark analysis of the Kaplan–Meier curves of cardiovascular events showed that there was no significant difference between the two pre-PCI groups during the first 1.5 years (*χ*^2^ = 1.36, *P* = .244, Fig. 2B). However, significant difference was found between the two post-PCI groups during the first 1.5 years (*χ*^2^ = 8.21, *P* = .004, Fig. 2D). Additionally, as also shown in Table 2, adjusted HR of cardiovascular events between the two post-PCI groups was significantly higher during the first 1.5 years (adjusted HR = 9.99, 95%CI = 1.99–50.13, *P* = .005), but lower and of no statistical difference from the first 1.5 years onwards (adjusted HR = 0.82, 95%CI = 0.26–2.59, *P* = .733). The secondary endpoints are also shown in Table 2, despite an inability of calculating several HR values due to the small numbers of events.

## 4 Discussion

To my knowledge, this is the first report about the prognostic value of Ang-2 in patients with CHD after elective PCI. This study found that the patients who had high serum Ang-2 concentrations of post-PCI, but not pre-PCI, had a significantly higher risk of cardiovascular events within the first 1.5 years after PCI.

Clinical studies have shown that peripheral blood Ang-2 concentrations are elevated in patients with CHD and are associated with the severity of coronary artery stenosis.^[17–19]^ Ang-2 expression is significantly upregulated in ischemic or necrotic myocardium.^[20,21]^ It has been demonstrated that the upregulation of Ang-2 as a result of hypoxia occurs widely in endothelial cells, both in vitro and in vivo.^[22]^ Recently, a previous study similar to this study found that decreased levels of plasma Ang-1, instead of Ang-2, can independently predict the development of 1-year MACE in patients with STEMI.^[23]^ Differing from this study, their research subjects were STEMI patients and the samples for Ang-2 measurement were collected only before PCI. The possible explanations for the differences in results of these two studies may include the following: Ang-2 is expressed weakly by resting endothelium but is highly upregulated following an inflammatory response.^[2]^ In the acute period of myocardial infarction, especially in STEMI, the predominant inflammatory response peaks after 24 to 28 h and leads to a significant release of Ang-2.^[24]^ In the subacute period, Ang-2 wanes gradually. In contrast, the expression of Ang-1 remains relatively constant at all times in the ischemic process.^[21]^ Therefore, the acute phase response of Ang-2 may overestimate the severity of coronary heart disease, which may be the reason why the previous study only found Ang-1 to be a MACE predictor after PCI. Whether the post-PCI Ang-2 can also predict MACE in patients of STEMI remains to be studied. Given the fact that PCI often leads to periprocedural myocardial injuries (PMI) due to procedural complications such as distal embolization and side-branch occlusion,^[25]^ the inflammatory response caused by the intervention itself is non-negligible. Overall, post-PCI serum Ang-2 levels were significantly lower than pre-PCI levels. However, there were a portion of patients whose Ang-2 levels decreased only a little or even increased after PCI. Therefore, periprocedural myocardial injury may be partly responsible for high post-PCI Ang-2 levels, which contributes to poor prognosis after PCI. Our blood samples were collected within 24 to 48 h after PCI, which may be too soon after PCI. Given the dynamic changes of Ang-2 levels over time after PCI,^[19]^ further studies are required to explore the possibility that postponing the blood extraction time to 1 week or even longer until the post-PCI Ang-2 level becomes steady would enhance its prediction capability.

In this study, the high pre-PCI Ang-2 levels was not able to predict the occurrence of cardiovascular events in the manner that post-PCI Ang-2 levels were able to. Possible explanations for this difference may include the following: First, this study is a single center design with a relatively small sample size. Enlarging the sample size may increase the accuracy of the results. Second, the level of Ang-2 before PCI may merely reflect ischemic severity of coronary heart disease. Since the patients had coronary artery stenosis at varying levels, the individual PCI treatments may have differently relieved the ischemic status. Therefore, the post-PCI Ang-2 levels may indicate the heart condition after the improvement of ischemia. Third, serum Ang-2 levels are also closely related to renal function^[26]^ and significantly increase after acute kidney injuries.^[3]^ It is well known that patients with contrast-induced nephropathy (CIN) after PCI are more likely to have adverse cardiovascular outcomes.^[27]^ Therefore, the occurrence of CIN that lead to the increased Ang-2 levels may partly explain why the post-PCI Ang-2 levels was of more prognostic value for cardiovascular events than the pre-PCI Ang-2 levels.

The value of Ang-2 in predicting adverse cardiovascular events has been widely reported. In acute myocardial infarction complicated by cardiogenic shock, high levels of Ang-2 are independently associated with mortality.^[3]^ In chronic heart failure, Ang-2 levels can be used to predict 1-year outcomes.^[4]^ Among patients with hypertension, elevated levels of Ang-2 are predictive of myocardial infarction.^[28]^ Ang-2 serum levels have been reported to be elevated in abdominal aortic aneurysm and associated with an increased risk of cardiovascular mortality in older men.^[5]^ In patients with chronic kidney disease, Ang2 levels can predict MACE and all-cause mortality.^[7]^ In the general population, high levels of circulating Ang2 and Tie-2 soluble receptors are associated with a greater risk of mortality.^[6]^ But, to date, we have not found any data on the role of Ang-2 in predicting cardiovascular events in patients with CHD after elective PCI.

Angiogenesis and inflammation are two highly interrelated processes.^[2]^ Ang-2, an important regulator of angiogenesis, can also influence inflammation and has been shown to possess pro-atherosclerotic effects.^[12]^ Ang-2 is able to sensitize endothelial cells towards TNF-α, which can stimulate the upregulation of related adhesion molecules.^[11]^ This would encourage the migration of monocytes into the arterial wall through the endothelium. Activated monocytes release proteolytic enzymes, such as matrix metalloproteases that promote extracellular matrix degradation and make the plaque vulnerable.^[29]^ In vivo, angiopoietin-2 blocking antibodies can reduce early atherosclerotic plaque development in mice.^[16]^ Additionally, Ang-2 may enhance the formation of new vessels within the plaque and accelerate its progress.^[15]^ Ang-2 counteracts the anti-inflammatory and anti-arteriosclerosis effect of Ang-1,^[30,31]^ which may severely impede the endothelial healing process. Hence, these effects may form a vicious circle, resulting in poor prognosis after PCI.

It is worthy to note that in some patients with recurrent severe angina, it is not observed of any restenosis artery through the repeat coronary angiography, which is probably due to endothelium-dependent coronary microvascular dysfunction.^[32]^ According to this study, patients with post-PCI Ang-2 levels higher than the median value had a significant increase in the risk of cardiovascular events during the first 1.5 years after PCI compared with patients whose post-PCI Ang-2 levels were lower than the median value. These findings will have high clinical potential, once the results are further confirmed by large-scale prospective studies. However, in this study, the prognostic value is limited from the first 1.5 years onwards. As mentioned before, Ang-2 is a very complex factor which participates in the regulation of many physiological and pathological processes. The short duration between intervention and blood extraction after PCI might have caused post-PCI Ang-2 levels to be influenced by factors, such as myocardial damage, acute kidney injury, inflammatory response, and electrolyte disturbance, which might be the partly reason why it could better predict the cardiovascular events in the short to medium term rather than the long term. Additionally, other factors, such as dietary changes, smoking, or new-onset comorbidity, may also compromise the prognostic value of Ang-2 in the long term. Therefore, regular monitoring of Ang-2 levels after discharge seems to be necessary and helpful. Patients will have better outcome when the cardiovascular events are identified earlier than its onset and prevented in advance.

However, our study has some limitations. First, it is limited by its single center design and relatively small sample size. In order to both include cumulative cardiovascular events as much as possible and show the short to medium term prognostic value, we chose 1.5 years as the cut-off point for landmark analysis, which may have caused a certain degree of bias. Second, we did not routinely monitor the change of serum creatinine and troponin levels after PCI unless there were clinical indications, which makes the relationship between the occurrence of PMI or CIN and post-PCI Ang-2 levels completely unknown. Additionally, other clinical factors such as drugs and disease stage of individuals have also not been analyzed. Third, we observed no occurrence of cardiac death, probably because our study subjects excluded the patients of acute myocardial infarction. Therefore, studies on a larger sample size with a higher number of endpoints should be performed to further investigate this issue.

## 5 Conclusion

High Ang-2 levels of post-PCI can predict the occurrence of cardiovascular events in the short to medium term.

## Author contributions

**Conceptualization:** Lang Li, Chun Gui.

**Data curation:** Wen Jian, Cheng-Qiang Wu, Chun Gui.

**Formal analysis:** Wen Jian, Chun Gui.

**Funding acquisition:** Chun Gui.

**Investigation:** Wen Jian, Xiao-Min Wei, Chun Gui.

**Methodology:** Chun Gui.

**Project administration:** Lang Li, Chun Gui.

**Resources:** Lang Li, Chun Gui.

**Software:** Wen Jian, Cheng-Qiang Wu, Chun Gui.

**Supervision:** Chun Gui.

**Validation:** Lang Li, Chun Gui.

**Visualization:** Wen Jian, Chun Gui.

**Writing – original draft:** Wen Jian.

**Writing – review & editing:** Wen Jian.
